# An antibacterial platform based on capacitive carbon-doped TiO_2_ nanotubes after direct or alternating current charging

**DOI:** 10.1038/s41467-018-04317-2

**Published:** 2018-05-24

**Authors:** Guomin Wang, Hongqing Feng, Liangsheng Hu, Weihong Jin, Qi Hao, Ang Gao, Xiang Peng, Wan Li, Kwok-Yin Wong, Huaiyu Wang, Zhou Li, Paul K. Chu

**Affiliations:** 1 Department of Physics and Department of Materials Science and Engineering, City University of Hong Kong, Tat Chee Avenue, Kowloon, Hong Kong China; 2 CAS Center for Excellence in Nanoscience, Beijing Key Laboratory of Micro-nano Energy and Sensor, Beijing Institute of Nanoenergy and Nanosystems, Chinese Academy of Sciences, Beijing, 100083 China; 3 School of Nanoscience and Technology, University of Chinese Academy of Sciences, Beijing, 100049 China; 4 Department of Applied Biology and Chemical Technology and the State Key Laboratory of Chirosciences, The Hong Kong Polytechnic University, Hung Hom, Kowloon, Hong Kong China; 5 Research Center for Biomedical Materials and Interfaces, Shenzhen Institutes of Advanced Technology, Chinese Academy of Sciences, Shenzhen, 518055 China

## Abstract

Electrical interactions between bacteria and the environment are delicate and essential. In this study, an external electrical current is applied to capacitive titania nanotubes doped with carbon (TNT-C) to evaluate the effects on bacteria killing and the underlying mechanism is investigated. When TNT-C is charged, post-charging antibacterial effects proportional to the capacitance are observed. This capacitance-based antibacterial system works well with both direct and alternating current (DC, AC) and the higher discharging capacity in the positive DC (DC+) group leads to better antibacterial performance. Extracellular electron transfer observed during early contact contributes to the surface-dependent post-charging antibacterial process. Physiologically, the electrical interaction deforms the bacteria morphology and elevates the intracellular reactive oxygen species level without impairing the growth of osteoblasts. Our finding spurs the design of light-independent antibacterial materials and provides insights into the use of electricity to modify biomaterials to complement other bacteria killing measures such as light irradiation.

## Abstract (web summary)

Bacteria are known to be sensitive to electrical interactions with the environment. Here, the authors report on a study into how the antibacterial properties of carbon-doped titania nanotubes are affected by capacitance after charging with direct and alternating currents.

## Introduction

Common biomedical materials such as titanium, titania, and many types of polymers do not have inherent antibacterial properties^1^, but bacterial resistance is essential in many clinical applications. Besides common strategies such as bulk addition^2^ and surface modification^3^ using antibiotics, introduction of agents such as Ag, Au, and graphene to the materials can produce antibacterial properties, and the role of electron transfer between the modified surface and bacteria has been suggested^4–11^. In fact, electron transfer is a crucial step in many bacterial activities. For instance, by means of electron transfer, bacteria complete respiration on the cell membrane to supply energy for cell growth, proliferation, and maintenance^12–14^ and disturbing electron transfer in bacteria can raise the production of reactive oxygen species (ROS) to hinder growth^15^. Another strategy for antibacterial functionalization is to create surface charges. Van der Mei et al. have reported that a positively charged carbon surface can reduce the viability of bacteria^16^. Positive or negative surface charges have also been found to promote the antibacterial efficiency of chitosan and inhibit adherence of Gram-negative bacteria on polymeric materials^17–20^. Further mechanistic studies indicate that surface charges can disrupt the membrane potential of bacterial cells producing irreversible damage in the membrane structure^21, 22^. Nonetheless, materials decorated with positive charges can only disinfect bacteria in a very short term and they are not yet effective in antibacterial applications.

Tian et al. have recently discovered that the ZnO/Ag nanobrushes charged by a triboelectric nanogenerator can exhibit sustained antibacterial effects even after the power supply has been turned off^23^. The post-charged samples can sterilize bacteria and this phenomenon is independent of electroporation of the substrates during electrical charging^24, 25^. However, it is not clear yet whether the post-charging disinfection is an individual phenomenon or generally related to the capacitance of materials. In addition, the underlying mechanism has not been explored systematically.

In this study, the post-charging antibacterial properties of capacitive materials are investigated. Titania nanotubes doped with carbon (TNT-C) predesigned with different capacitances are subjected to both direct and alternating current (DC, AC) to explore the post-charging antibacterial effects. Owing to the larger discharging capacity, the positive DC (DC+) charging mode shows better bacteria killing effects than AC charging. Besides, the capacitance-based platform can effectively prevent biofilm formation by means of cyclical charging. Extracellular electron transfer (EET) between the bacteria and charged TNT-C impairs the morphology of bacteria and induces ROS burst in the bacteria, contributing to post-charging death of bacteria, but the growth of osteoblasts is not affected. This is a systematic study on the post-charging antibacterial properties of biomaterials with tuneable capacitance and the results provide insights into the design and construction of biomaterials with antibacterial functions.

## Results

### Sample characterization

**Fig. 1:**
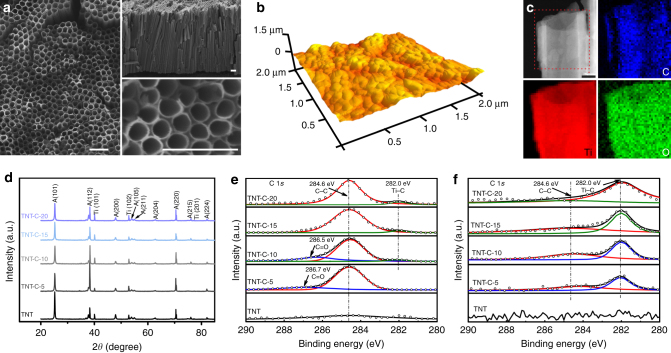
Representative sample characterization results. **a** SEM image of TNT-C-15 with the insets showing the corresponding enlarged and cross-sectional images (Scale bar = 500 nm). **b** AFM image of TNT-C-15 showing the surface morphology. **c** STEM-EELS maps of C, Ti, and O of TNT-C-15 (Scale bar = 50 nm). **d** XRD patterns of the TNT and TNT-C samples. **e**, **f** High-resolution C 1*s* spectra of the TNT and TNT-C samples acquired **e** from the surface and **f** after sputtering for 6 min. The sputtering rate is approximately 21 nm min^−1^ referenced to SiO_2_

The optical images of the TNT and TNT-C samples are shown in Supplementary Fig. 1. TNT is yellow and the color of the TNT-C samples changes from gray to dark brown when the heating rate is increased from 5 °C min^−1^ to 20 °C min^−1^ during annealing, suggesting increasing amounts of C. The scanning emission microscopy (SEM) images in Fig. 1a reveal that TNT-C-15 (heating rate at 15 °C min^−1^ during annealing) fabricated on Ti has an outer diameter of 160 nm, wall thickness of 25 nm, and nanotube length of 10 μm. Compared to TNT, the overall morphology of TNT-C for heating rates no more than 15 °C min^−1^ does not differ significantly, while some of the nanotubes are broken if the heating rate is 20 °C min^−1^ (Supplementary Fig. 2). On the basis of the atomic force microscopy (AFM) images in Fig. 1b and Supplementary Fig. 3, the roughness of the TNT-C samples (bar chart in Supplementary Fig. 3) is similar and slightly less than that of TNT. Scanning transmission electron microscopy (STEM) and electron energy loss spectroscopy (EELS) show that C is uniformly distributed in the entire tube walls (Fig. 1c). X-ray diffraction (XRD) discloses dominant diffraction peaks at 2*θ* = 25.3° (101), 48.0° (200), and 70.3° (220) from all the samples, indicating the formation of anatase TNT and (101), (102) and (201) phases of Ti beneath (Fig. 1d). The TNT and various TNT-C samples show the same peaks confirming that the crystallinity is not altered by C doping.

X-ray photoelectron spectroscopy (XPS) is employed to characterize the chemical states of the various samples. The XPS survey scan (Supplementary Fig. 4a) shows that apart from Ti and O in TNT, a strong C 1*s* peak at 284 eV is detected from the TNT-C samples and the intensity of the C 1*s* peak increases with heating rates. During annealing in air, the residual ethylene glycol in the as-anodized nanotubes is converted into CO_2_ and released to the air flow and therefore C is not detected from the TNT sample. On the contrary, the residual ethylene glycol is carbonized and C is doped in the nanotubes during annealing in Ar. As the gas pressure in the nanotube is larger than that on the surface^26^, the nanotubular space provides a higher pressure environment to enhance the decomposition of ethylene glycol, leading to subsequent formation of TNT-C during annealing in Ar. The C concentration on the surface of the TNT-C samples is about 25% and drops to 1.9, 3.0, 5.8, and 6.9% after sputtering for 8 min (sputtering rate of ~21 nm min^−1^ referenced to SiO_2_) for TNT-C-5, TNT-C-10, TNT-C-15 and TNT-C-20, respectively (Supplementary Fig. 4b). This is because the residual ethylene glycol underneath partially volatilizes during the heating process and the surface layers have a larger C concentration than the bottom part of the nanotubes. The high-resolution C 1*s* spectra are obtained from the surface and after sputtering for 6 min (Fig. 1e, f). On the surface, *sp*^2^ C–C (284.6 eV) is the dominant component and the shoulders at binding energies of 282.0, 286.5 eV, and 288.9 eV are related to C–Ti, C–O and C=O, respectively (Fig. 1e)^27^. After sputtering for 6 min, the peaks associated with C=O (288.9 eV) and C–O (286.5 eV) disappear, whereas the intensity of the C–C peak (284.6 eV) decreases and that of the C–Ti peak (282.0 eV) increases notably (Fig. 1f). The transition from C–C to C–Ti can be explained by that carbon partially substitutes for oxygen in TiO_2_ forming Ti–C bond (TiO_*x*_C_2−*x*_)^27, 28^. The Ti 2*p* XPS spectra acquired from the surface and after sputtering for 6 min suggest that TiO_2_ is partially reduced to Ti–C, Ti^2+^, and Ti^3+^ (Supplementary Fig. 4c and 4d). In combination with the STEM results, it can be concluded that different amounts of carbon are incorporated into the titania nanotubes using the simple one-step annealing process.

### Capacitive analysis

**Fig. 2:**
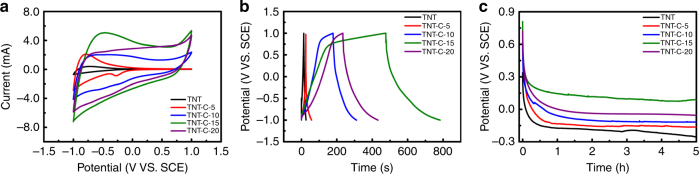
Electrochemical performance. **a** CV and **b** GCD curves acquired at a scanning rate of 100 mV s^−1^ and current density of 2.5 mA cm^−2^, respectively. **c** Potential of samples with time after charging to 1 V

The capacitive properties are evaluated using a three-electrode configuration with the luria broth (LB) medium as the electrolyte. Figure 2a presents the cyclic voltammetry (CV) curves of the TNT and different TNT-C samples at a scanning rate of 100 mV s^−1^. For TNT and TNT-C-5, no obvious electrochemical double-layer (EDL) is observed, but the curves of TNT-C-10, TNT-C-15, and TNT-C-20 present a typical EDL capacitive behavior. As the heating rate of TNT-C is increased from 5 °C min^−1^ to 15 °C min^−1^, the current in the CV profiles increases gradually and the maximum areal capacitance is observed from TNT-C-15. However, the specific capacitance decreases when the heating rate is increased to 20 °C min^−1^, possibly due to detachment and damage of the nanotubes as shown in Supplementary Fig. 2. The difference in the CV shape between our results and previous studies is possibly caused by the different scanning voltage ranges^29^. A similar capacitance tendency is observed from the corresponding galvanostatic charging-discharging (GCD) plots (Fig. 2b) at a current density of 2.5 mA cm^−2^ confirming the maximum capacitance of TNT-C–15. The potential curves with time after full charging are presented in Fig. 2c. The potentials on all the samples exhibit a sharp drop during the first hour and the curves are nearly horizontal afterwards. Comparing the steady-state voltages, TNT-C-15 is 0.2 V larger than that of the reference electrode and better than the other samples. The results corroborate that TNT-C-15 has the highest capacitance among the experimental groups.

### Capacitance-dependent antibacterial effects

**Fig. 3:**
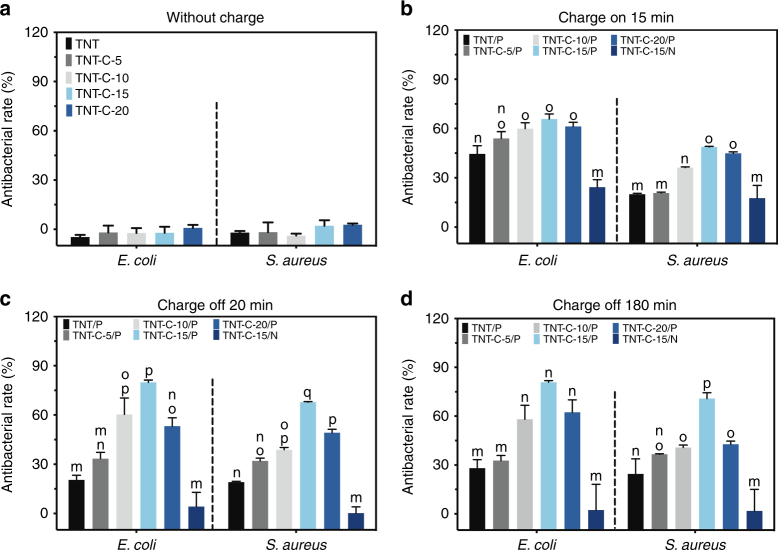
Antibacterial effects under different conditions. **a**, **b** Antibacterial rates of various samples **a** without charging and **b** during DC charging for 15 min. **c**, **d** Post-charging antibacterial rates of charged samples after **c** 20 min and **d** 180 min of bacteria incubation. All error bars = standard deviation (*n* = 3). P denotes DC+ charging and N denotes DC– charging. Significant differences between groups are marked with different letters (m–q, *P* < 0.05, SNK test in ANOVA)

In a next step, the samples with different capacitances are subjected to DC charging and their performance against micro-organisms is studied. Since Ti is as good as the petri dish in supporting bacteria growth and proliferation (Supplementary Fig. 5), it is used as the basic control to determine the antibacterial rates. As shown in Fig. 3a, none of the TNT or TNT-C samples exhibit any adverse effects on bacteria growth without charging thereby excluding the possibility that the antibacterial properties of charged TNT-C arise from the nanotube structure or C doping. Different TNT-C samples show different antibacterial efficiency during charging, with TNT-C-15 being the best one killing 50% of *Staphylococcus aureus* and 65% of *Escherichia coli* (Fig. 3b). More importantly, some of the charged samples continue to kill bacteria after the DC+ power has been turned off, with TNT-C-15 killing 80% of the *E. coli* and 68% of the *S. aureus* within 20 min (Fig. 3c). The antibacterial rates for these two bacteria strains are improved only slightly after extending the culture time from 20 min to 180 min (Fig. 3d), revealing that the antibacterial effects of the charged samples occur during early contact. To confirm the universal antibacterial effects of the capacitive TNT-C, TNT-C-15, which shows the best results in post-charging is tested with two additional strains of bacteria (*Staphylococcus epidermidis* and *Pseudomonas aeruginosa*). Supplementary Fig. 6 demonstrates that after charging with DC+ for 15 min, TNT-C-15 kills 75% of the *S. epidermidis* and 45% of the *P. aeruginosa* within 20 min.

Among the various TNT-C samples, TNT-C-15 with the highest capacitance shows the best bacteria killing rates implying that the post-charging antibacterial properties of TNT-C samples is capacitance dependent. Meanwhile, Gram-negative bacteria are more susceptible to the capacitance-dependent system than Gram-positive bacteria and it can be ascribed to the thinner membrane of Gram-negative bacteria^30, 31^. It should also be noted that the antibacterial effects of the TNT-C-15 after negative DC (DC–) charging are negligible (Fig. 3c, d). The reason is most likely that the negatively-charged surface repels bacteria with negative surface charges and bacteria survive due to the less surface contact. Moreover, as the TNT-C samples can undergo cyclical charging without losing capacitance (Supplementary Fig. 7), better antibacterial effects can be anticipated by recycling. Our further studies demonstrate that 93% of Gram-negative bacteria (*E. coli* and *P. aeruginosa*) and 88% of Gram-positive bacteria (*S. aureus* and *S. epidermidis*) can be sterilized after recharging the TNT-C-15 sample in the DC+ mode (Supplementary Fig. 8). After another cycle of DC+ charging, this platform can achieve a bacterial killing efficacy of over 90% for all the four genera of bacteria, thus boding well for clinical application. The accumulative antibacterial effects by recharging process is not very far from clinic with reference to the widespread use of implantable cardiac pacemaker. Furthermore, theoretical calculation^32^ and in vivo studies show that it is feasible to generate energy via implantable energy harvesters based on oxidation of internal glucose^33^ or body motions^34–36^. Future research on identifying more effective doping methods and materials may produce sufficient capacitance with one charging cycle to yield the desirable antibacterial efficiency.

### Antibacterial effects in DC and AC charging modes

**Fig. 4:**
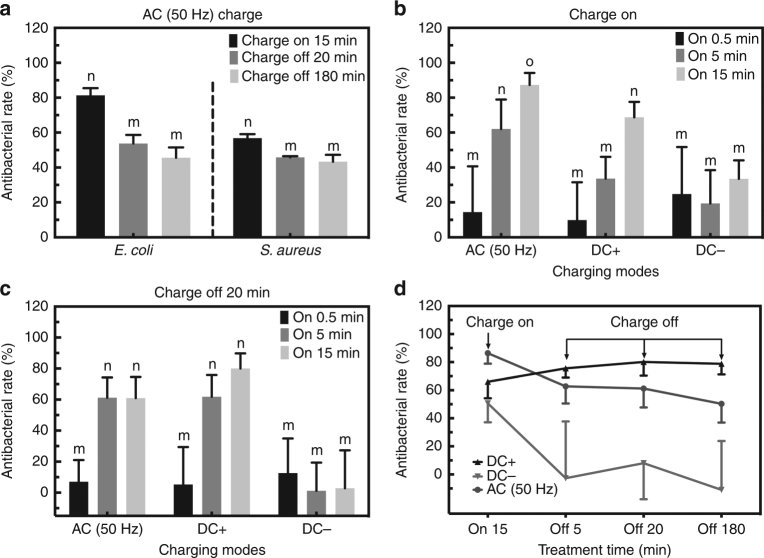
Antibacterial effects in different charging modes. **a** Antibacterial effect on *E. coli* and *S. aureus* triggered by AC. **b** On-charging antibacterial effects of TNT-C-15 on *E. coli* during the charging process. **c** Post-charging antibacterial effects of TNT-C-15 on *E. coli* after charging for different time. **d** Overall antibacterial curves of TNT-C-15 on *E. coli* triggered by AC, DC+ and DC–. All error bars = s.d. (*n* = 3). Significant differences between groups are marked by different letters (m–o, *P* < 0.05, SNK test in ANOVA)

AC is also applied to explore if the capacitance-dependent antibacterial effects depend on the charging modes. After 15 min of AC (50 Hz) charging, about 80% of *E. coli* and 60% of *S. aureus* cultivated on TNT-C-15 are killed (Fig. 4a). The post-charging antibacterial efficiency of AC charged TNT-C-15 is about 55% for *E. coli* and 50% for *S. aureus* within 20 min and does not go up much within 180 min. Besides, the frequency of AC has no influence on the post-charging antibacterial efficiency (Supplementary Fig. 9).

The antibacterial performance of the DC and AC systems is compared in details. When the platform is being charged, the antibacterial efficiency increases with charging time and reaches 70–90% for both the AC and DC+ groups. However, the antibacterial efficiency of the DC− group is <30% and changes very little with charging time (Fig. 4b). After charging, both the AC and DC+ groups retain the post-charging antibacterial ability but the DC− groups show an antibacterial rate below 10% (Fig. 4c). As the charging time is increased from 0.5 to 15 min, the post-charging antibacterial rate of the DC+ group increases from about 5–80%. Meanwhile, the post-charging antibacterial rate of the AC group only reaches 60%, meaning that DC+ is superior to AC in triggering the post-charging antibacterial effects. The time-dependent post-charging antibacterial rates of the AC, DC+, and DC− groups are displayed in Fig. 4d. The post-charging antibacterial rates change insignificantly from 5 to 180 min verifying that the antibacterial effects after charging occur during early contact.

### Morphological changes of bacteria

**Fig. 5:**
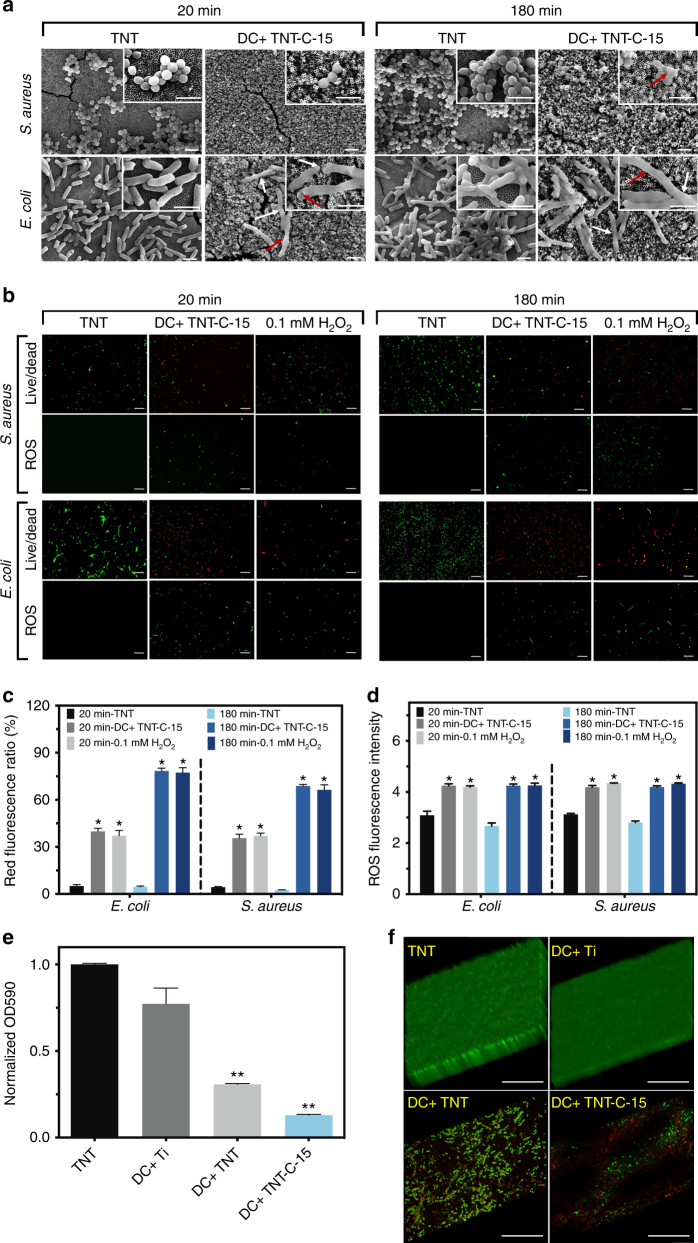
Physiological changes of bacteria. **a** SEM images of *S. aureus* and *E. coli* on TNT control and the DC+ charged TNT-C-15 (Scale bar = 2 μm). **b** Live/dead and ROS staining images of *S. aureus* and *E. coli* treated with TNT control, DC+ charged TNT-C-15, and 0.1 mM H_2_O_2_ (Scale bar = 20 μm). **c** Quantitative analysis of the live/dead staining results by flow cytometry. **d** Quantitative analysis of the ROS fluorescence intensity of treated bacteria by flow cytometry. **e**, **f** Anti-biofilm performances of various samples: **e** quantitative measurements after crystal violet staining and **f** 3D morphology of the fluorescently stained biofilms (Scale bar = 50 μm) (*denotes *P* < 0.05 and **denotes *P* < 0.01 compared with TNT group, Student *t* test). All error bars = s.d. (*n* = 3)

The bacteria treated with DC+ charged TNT-C-15 are further examined by SEM and the typical images are displayed in Fig. 5a. The bacteria cultured on TNT without charging serve as the control and the density of both *S. aureus* and *E. coli* on the charged TNT-C-15 is significantly smaller than that in the control group. Some of the *S. aureus* and *E. coli* on the DC+ charged TNT-C-15 exhibit an abnormal shape with damaged membranes (red arrows). For *E. coli*, the morphological change of bacteria filamentation is even found (white arrows), which is often observed as a result of bacteria response to various stresses, including oxidative stress and DNA damage^37^.

### Physiological changes of bacteria

The bacteria treated with DC+ charged TNT-C-15 and uncharged TNT are fluorescently stained to examine the viability and intracellular ROS levels. A concentration of 0.1 mM H_2_O_2_ is employed as the ROS positive reference as H_2_O_2_ is capable of sterilizing bacteria by triggering oxidative stress^38^. Figure 5b shows that *S. aureus* and *E*. *coli* grow well on TNT with green staining, whereas more than half of the bacterial cells on the DC+ charged TNT-C-15 are stained red signifying viability reduction. As shown by the quantitative results in Fig. 5c, the real-time ratios of dead *E. coli* are 40% and 75% at post-charging time points of 20 and 180 min, and the corresponding ratios of dead *S*. *aureus* are 35% and 65%, respectively. The antibacterial efficiency calculated from the live/dead staining results are comparatively lower than that of the colony forming unit (CFU) counting results (Fig. 3) and it can be explained by the viable but non-culturable state of the treated bacteria^39^. The *S. aureus* and *E. coli* on the DC+ charged TNT-C-15 show positive ROS signals similar to the 0.1 mM H_2_O_2_ group, revealing that oxidative stress is induced during the post-charging antibacterial process (Fig. 5b). Quantitatively, obvious ROS signal increase is detected from the bacteria on the DC+ charged TNT-C-15 consistent with the fluorescent images (Fig. 5b), suggesting that the charged TNT-C-15 produces instant and rapid oxidative stress even at 180 min post-charging (Fig. 5d). The membrane potentials of the bacteria decrease significantly in a similar way using DC+ charging of TNT-C-15 and 0.1 mM H_2_O_2_ (Supplementary Fig. 10). As an indicator of membrane integrity, the membrane potential is usually smaller in bacteria with damaged membranes and our results provide evidence that oxidative stress induced by the capacitive sample kills bacteria by destroying the membrane.

### Anti-biofilm assessments

As rechargeable platforms, the Ti, TNT and TNT-C-15 samples are further compared for their 48 h anti-biofilm performance by cyclical charging. For reference, the uncharged TNT is cultured with bacteria for the same period of time and serves as the control group. *E. coli* growth is significantly reduced on the charged TNT-C-15 vs. TNT group based on the normalized crystal violet absorbance (Fig. 5e). The better anti-biofilm ability of charged TNT-C-15 than charged Ti further verifies the importance of the capacitive properties in impeding the attachment and proliferation of *E. coli*. Furthermore, a positive correlation between the anti-biofilm effect and capacitance is revealed (charged TNT vs. charged TNT-C-15). Fluorescent staining and crystal violet results show that uniform and dense biofilms (10–20 μm in thickness) are formed on the uncharged TNT and charged Ti, whereas the cell densities on DC+ charged TNT and TNT-C-15 are smaller. Specifically, DC+ charged TNT-C-15 shows very small and discrete bacteria colonies and many colonies are red suggesting dead bacteria (Fig. 5f). As verified by SEM (Supplementary Fig. 11), the charged TNT-C-15 group delivers the best anti-biofilm performance and the capacitance-based platform is effective in biofilm prevention by means of cyclical charging.

### Mechanism from the electrical aspect

**Fig. 6:**
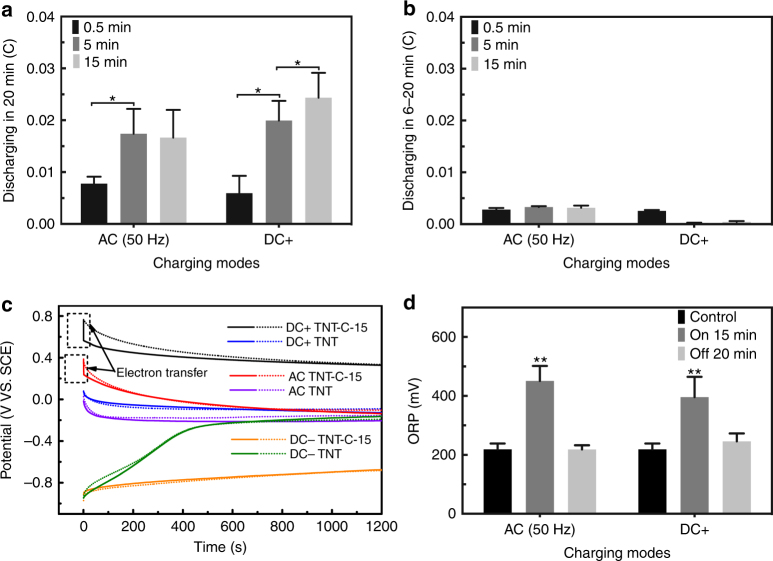
Post-charging antibacterial mechanism analysis. **a**, **b** Total discharging capacity of TNT-C-15 in **a** 0–20 min and **b** 6–20 min after AC/DC+ charging for 0.5, 5, and 15 min (*denotes *P* < 0.05, Student *t* test). **c** Potential curves of DC+ charged TNT-C-15, DC+ charged TNT, AC charged TNT-C-15, AC charged TNT, DC– charged TNT-C-15 and DC– charged TNT with solid/dashed lines denoting with/without bacteria, respectively. The pre-charging time is 15 min. **d** ORP in the LB medium during and after the charging process (**denotes *P* < 0.01 compared with the control group, Student *t* test). All error bars = s.d. (*n* = 3)

In order to determine the factors causing the post-charging antibacterial difference in the DC+ and AC groups, the corresponding discharging capacity of both groups is calculated for different pre-charging time. As shown in Fig. 6a, the discharging capacity in the AC group increases from 0.008 to 0.02 C when the charging time is increased from 0.5 to 5 min and it is maintained at 0.02 C when the pre-charging time is further increased to 15 min (Fig. 6a), suggesting that the sample can no longer be charged by AC in the 5~15 min charging process. In contrast, the discharging capacity of TNT-C-15 increases continuously from 0.005 to 0.025 C after DC+ charging from 0 to 15 min (Fig. 6a). The discharging performance of the DC+ and AC groups is consistent with the respective antibacterial efficiency. Figure 6b shows that the discharging capacity in 6–20 min is negligible for both groups. This is consistent with the findings discussed above that early interactions between the bacteria and charged samples are crucial to the post-charging antibacterial progress.

The interaction between the bacteria and samples is further evaluated electrochemically. Figure 6c depicts the discharging curves of TNT and TNT-C-15 with/without bacteria added to the electrolyte. Among the TNT-C groups, a quick potential drop is observed from the DC+ and AC charged TNT-C-15 when bacteria are added to the electrolyte (black and red lines shown in Fig. 6c) due to the instant EET of bacteria in contact with the samples. This contact-induced EET appears to be responsible for post-charging bacteria killing. Contrarily, no difference is observed when bacteria are added to the DC− charged TNT-C-15 (orange lines shown in Fig. 6c). This can be attributed to the inherently negative membrane potential of the bacteria, which can lose electrons more easily in contact with a positively charged surface rather than a negatively charged surface. On the other hand, compared to TNT (blue, purple and green lines shown in Fig. 6c), it takes a longer time for the TNT-C-15 systems (black, red and orange lines shown in Fig. 6c) to reach the open circuit potential, thus verifying the capacitance-based antibacterial behavior.

### Mechanism in terms of physicochemical effects

As shown in Fig. 6d, the oxidation-reduction potential (ORP) of the LB medium during charging for 15 min increases significantly, but the charged samples can no longer produce a post-charging ORP increase. The significant oxidative stress in the on-charging LB may be responsible for the antibacterial effect during the charging process but after charging, little oxidative stress is detected from the medium showing that the post-charging antibacterial behavior of TNT-C-15 is surface dependent. In addition, there is no significant difference in the pH of the medium treated by different measures thereby excluding the pH-dependent antibacterial mechanism (Supplementary Fig. 12).

### Biocompatibility assessments

**Fig. 7:**
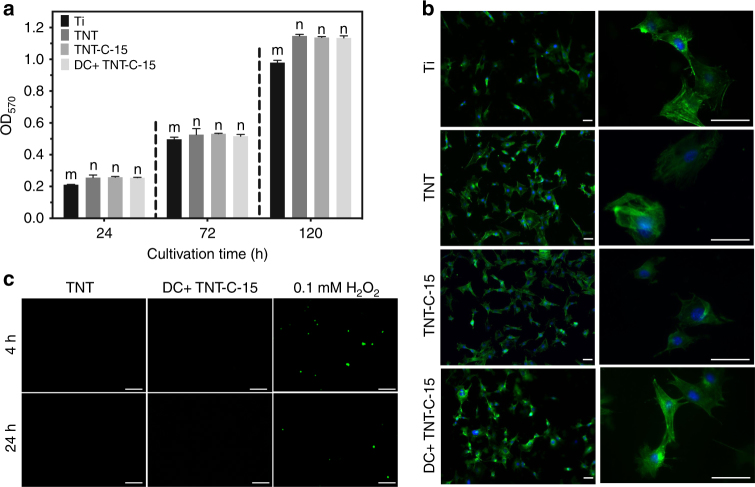
Biocompatibility assessments. **a** Quantitative determination of the cell viability on various samples (significant differences between groups are marked by m and n, *P* < 0.05, SNK test in ANOVA). **b** Cell morphology of MC3T3-E1 osteoblasts cultivated on different samples for 24 h (Scale bar = 50 μm). **c** ROS observation of MC3T3-E1 osteoblasts cultured on different samples for 4 h and 24 h (Scale bar = 100 μm). All error bars = s.d. (*n* = 3)

For biomedical implants, not only the antibacterial performance but also the biocompatibility is crucial. In this respect, the 3-(4,5-dimethylthiazol-2-yl)-2,5-diphenyl tetrazolium bromide (MTT) assay is carried out to gauge the cytotoxicity of the post-charging samples. As Ti has been widely used in various types of bone-anchored reconstructions^40^ and growth curves of osteoblasts on petri dish and Ti show little difference (Supplementary Fig. 13), Ti is used as the basic control group to evaluate the compatibility of the various platforms. The results demonstrate that the MC3T3-E1 osteoblasts growing on the DC+ charged TNT-C-15 exhibit no difference in the viability compared to the uncharged TNT or TNT-C-15, and even better than the Ti control group (Fig. 7a). The cytoskeleton of MC3T3-E1 osteoblasts is also determined by fluorescent staining. As illustrated in Supplementary Fig. 14, most of the cells in the Ti group retain the original round shape after culturing for 4 h, whereas those on the TNT, TNT-C-15, and charged TNT-C-15 exhibit some filopodia. After cell culturing for 24 h, the cells spread well on all the sample surfaces (Fig. 7b). These results suggest that TNT-C-15 is a suitable milieu for osteoblasts and electrical charging causes no side effects to cell attachment and proliferation. After cell culturing, the DC+ charged TNT-C-15 group is compared with the TNT group and positive control group (0.5 mM H_2_O_2_) for the ROS levels of cells. Figure 7c reveals that different from bacteria, no detectable oxidative stress is exerted on the osteoblasts in the DC+ charged TNT-C-15 group.

The functionalized surface that can specifically disinfect bacteria but does not harm mammalian cells has been reported before^8, 41–43^. This phenomenon may stem from that the respiration chain, which modulates the electron transfer and ROS production lies in the intracellular organ mitochondrion in mammalian cells. Therefore, mammalian cells are less susceptible than prokaryotic bacteria to the external electricity. Meanwhile, the nanotube structure with a specific size effect^44, 45^ accounts for the enhanced viability of TNT and TNT-C-15. As a result, this post-charging system causes bacteria to die but supports the growth of osteoblast cells. The dual properties are highly desirable for medical implants.

## Discussion

TNT is well known for the photocatalytic antimicrobial properties but the wide bandgap of TNT limits its application to only UV irradiation and the photocatalytic bacteria killing process is restricted by the small electron transfer rate and high electron-hole recombination rate^46–49^. Moreover, photocatalytic bacteria killing is impractical in many in vivo applications. Herein, we demonstrate that TNT doped with C can sterilize bacteria without UV pre-irradiation and the charging-discharging process plays an important role. The post-charging antibacterial platform is demonstrated to be efficient enough to prevent biofilm formation by means of cyclical charging extending biomedical and even clinical applications.

The capacitive properties of the TNT-C samples can be tuned by a simple one-step annealing process of the as-anodized TNT. The residual ethylene glycol electrolyte after anodization acts as the C supplier during annealing and the concentration of C can be tailored by simply adjusting the heating rate to endow samples with different capacitances. C is evenly distributed in the nanotube structure during fabrication and C–C is converted to C–Ti controllably by adjusting the annealing conditions^50^. C incorporation into TiO_2_ alters the electrical properties^51^ and increases the ion adsorption capacity and surface area of materials^52, 53^ giving rise to higher capacitance. The amount of C in TNT-C-15 is larger than those in TNT-C-5 and TNT-C-10, and therefore, TNT-C-15 is better than TNT-C-5 and TNT-C-10 regarding to the capacitive performance. The C content in TNT-C-20 is slightly larger than that in TNT-C-15 but if the heating rate is 20 °C min^−1^, the nanotubes tend to be broken and detached from the substrate to undermine the capacitance. On the basis of the experimental evidence, TNT-C-15 is the optimal one among the TNT-C samples. Some previous studies have also shown that TNT-C has good electrochemical stability and double layer charge storage^53, 54^.

An important observation from our experiments is that the post-charging antibacterial effect is related to the electrical properties of the samples and it can be explained as follows. Among all the charged samples with different C contents, TNT-C-15 with the highest capacitance exhibits the best bactericidal effect. The DC+ charged TNT-C-15 is more effective than the AC charged one for killing bacteria (Fig. 4b, c), which is consistent with the discharging capacity result (Fig. 6a), suggesting that a capacitator which stores more charges can kill bacteria more easily. AC charging is also capable of producing post-charging properties, suggesting that the AC energy can be collected by TNT-C as well. In general, the antibacterial effects of TNT-C can be enhanced by increasing the sample capacitance^55, 56^ and optimizing the charging scheme.

Another interesting result is that the DC– charged samples possess little bacteria killing ability. No significant potential plunge is observed when bacteria are added to the DC– charged samples (Fig. 6c) compared to the DC+ or AC charged ones. This may be associated with the intrinsic negative membrane potential on the bacteria^18^, resulting in a repulsive force between the DC– charged surface and the inherently negatively charged bacteria membranes. This in turn hinders the bacteria-sample interaction and prevents bacteria from destruction. On the contrary, the DC+ charged TNT-C surface has a high affinity to bacteria cells, interacts immediately with the negatively-charged groups of acidic phospholipids in the cellular membrane, leads to the leakage of intracellular components, and consequently causes bacteria death^57^. Our results are in agreement with the advantage of positive charges over negative charges in bacterial killing observed previously^58^.

**Fig. 8:**
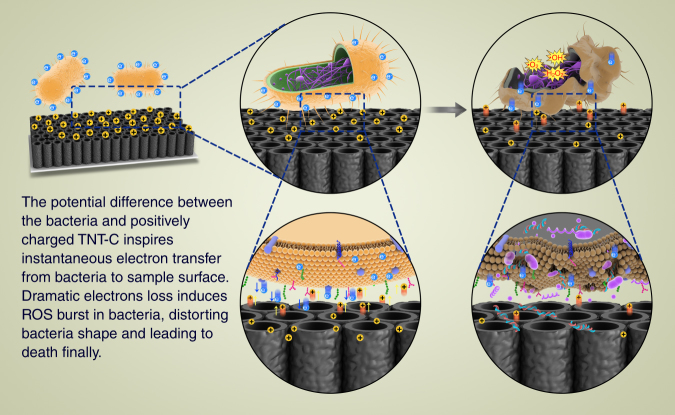
Diagram showing antibacterial mechanism. Proposed antibacterial process on DC+ charged TNT-C based on the experimental results

Figure 6c provides evidence of direct electron transfer between the bacteria and DC+/AC charged surface. The ORP and pH of the LB medium during post-charging bacteria cultivation are the same as those of fresh LB thus excluding any killing contribution from the medium (Fig. 6c). Intracellular over-production of ROS is observed from the bacteria on the DC+ charged surface but not detected from the uncharged control group (Fig. 5b, d) and so it plays an important role in the bactericidal process. As a common intracellular signaling molecule, ROS is usually produced along the respiratory chain with electrons steadily donated to O_2_. Generally, “oxidative stress” occurs when ROS production changes from adaptive to maladaptive^59^ and an elevated level of intracellular ROS is considered lethal to prokaryotic cells because oxidization of essential substances such as DNA, lipids, and proteins can cause overall cell damage and final death^60^. As stated above, EET takes place in contact with the charged TNT-C and electron transport along the respiratory chain is impeded to produce ROS^61–63^. Since TNT-C-15 has a larger capacitance, the electronic disturbance is stronger producing more ROS, damaging the bacterial membrane, and giving rise to better antibacterial effects. Basing on our analysis, Fig. 8 illustrates the possible antibacterial process of the charged TNT-C system. It is emphasized that the capacitance-dependent antibacterial platform described here is green and environmentally friendly and can be integrated with an implantable battery or self-powered system capable of harvesting energy in the human body^64^ to realize the tremendous clinical potential.

In conclusion, capacitive TNT-C samples with different C contents are fabricated by simple annealing and charged by different electrical modes to investigate the post-charging antibacterial process. Both the DC+ and AC charging schemes produce on-charging and post-charging antibacterial properties from TNT-C. TNT-C-15 charged by DC+ for 15 min shows the best post-charging bactericidal and anti-biofilm effects, which are attributed to the inherently superior capacitance and largest discharging capacity in contact with bacteria. A potential plunge is observed from the discharging curves of the DC+ and AC groups furnishing evidence about electron transfer between the bacteria and positively charged surface. On the basis of the electrical and biological analyses, electron transfer near the interface produces stress in the bacteria, elevates the intracellular ROS, deforms the morphology, produces bacteria death, and prevents biofilm formation. The antibacterial results and good compatibility with osteoblasts reveal the large potential of exploiting the capacitance of biomaterials as well as application of electrical currents to complement the light-dependent antibacterial properties of biomaterials.

## Methods

### Preparation of TNT-C and sample characterization

The titania nanotubes were prepared by electrochemical anodization of a Ti metal foil in an NH_4_F-ethylene glycol solution and the ordered TNT-C was fabricated by annealing the as-anodized titania nanotubes in Ar^29, 55, 65, 66^. The Ti foil with dimensions of 30 × 30 × 0.5 mm was ground with abrasive paper (from 400 to 2000-grit), cleaned ultrasonically in acetone, alcohol, and deionized water, and dried in nitrogen. Anodization was performed at 60 V for 1 h at room temperature (25 °C) in a conventional two-electrode cell with a DC power supply (IT6123, ITECH, Nanjing, China). The graphite and Ti foils serving as the cathode and anode, respectively, were separated by 1 cm and the electrolyte contained 0.55 g of ammonium fluoride, 5 mL of deionized water, and 95 mL of ethylene glycol. The anodized nanotubes were rinsed in 2 mL of deionized water for 5 s, dried in nitrogen for 5 s, and annealed at 500 °C for 180 min in a tube furnace in air (named TNT) or under flowing Ar (80 SCCM) at heating rates of 5, 10, 15 and 20 °C min^−1^ (labeled TNT-C-5, TNT-C-10, TNT-C-15 and TNT-C-20, respectively) to obtain different C concentrations. The residual ethylene glycol in the TNT served as the C source and an extra C precursor was not required. The annealed samples were sonicated in water for 20 min to remove nanowires from the top of the nanotubes. The samples were characterized by SEM (XL30, ESEM-FEG, Philips, Holland) and AFM (Veeco MultimodeV). XRD (Siemens D500, Philips, Netherlands) with Cu K_α_ irradiation (*λ* = 1.54184 Å) was conducted to determine the crystallinity of the samples at 30 kV and 10 mA. EELS was performed on the 200 kV field-emission STEM (JEOL JEM-2010F) equipped with the Gatan Tridiem electron energy loss spectrometer. The chemical states were determined by XPS (K-Alpha, Thermo Fisher Scientific, USA) with Al K_α_ radiation referenced to the Ar 2*p* peak at 242.4 eV (Supplementary Fig. 4e).

### Antibacterial analyses

The antibacterial activity of the sterilized samples was assessed with Gram-positive (*S. aureus*, 29213) and Gram-negative (*E. coli*, ATCC 25922) bacteria. In brief, the pure bacteria in LB were cultivated overnight in a rotating shaker at 37 °C, diluted to twice the volume and cultivated to a concentration of 2–3 × 10^9^ CFU mL^-1^ (OD_600_ = 0.3 for *S. aureus* and OD_600_ = 1.0 for *E. coli*). The bacteria solution with a concentration of 2–3 × 10^5^ mL^−1^ was prepared for the antibacterial test. The samples were immersed in 75% alcohol for 30 min for sterilization and dried in nitrogen before they were prepared on the anode of the reaction kettle. 10 mL of the bacteria solution were added to the reaction kettle prior to the application of external DC or AC currents (Supplementary Fig. 15, Supplementary Method 1). After the treatment, the bacteria were diluted and spread on agar plates for the CFU analysis. The charged samples were cleaned with phosphate-buffered saline (PBS) and put on the wells of a 6-well plate. 400 μL of the bacteria solution were spread on the surface and after a period of time, the bacteria were diluted and spread on agar plates for CFU analysis to determine the post-charging antibacterial effects. To confirm the post-charging antibacterial effects, TNT-C-15, which shows the best was tested with two additional strains of bacteria (*S. epidermidis* and *P. aeruginosa*, Supplementary Method 2). Besides, the accumulative antibacterial effect as well as anti-biofilm effect by recharging process was also measured (Supplementary Methods 3 and 4).

### Capacitance and discharging capacity of samples

The electrochemical properties of the samples were characterized using a three-electrode system on an electrochemical workstation (Zennium, Zahner, Germany) in LB to mimic the growth environment of bacteria. The sample (3 cm^2^), platinum wire, and saturated calomel electrode (SCE) served as the working electrode, counter electrode, and reference electrode, respectively. CV was carried out from −1 to 1 V at a scanning rate of 100 mV s^−1^ and GCD tests were performed at a constant charging current of 2.5 mA cm^−2^. A one-time discharging curve of the charged (charged to 1 V) sample was recorded for 5 h with the working electrode and counter electrode separated by 1 cm. On the basis of the antibacterial test and charge storage measurement, TNT-C-15 was selected to assess the discharging behavior after charging with DC and AC. After charging for different periods of time, the samples were immediately transferred to the electrochemical system to acquire the discharging curves. The recyclable capacitive properties of materials were also evaluated after the antibacterial process (Supplementary Method 5).

### Interactions between bacteria and charged samples

Using the aforementioned electrochemical platform, the electrical interaction between the bacteria and charged samples was tested on TNT and TNT-C-15 in the antibacterial and capacitance tests. After charging, the bacteria were added to the LB medium (with final concentration of 2–3 × 10^5^ CFU mL^−1^) and the cathode and anode were separated with a 0.22 μm membrane. The one-time discharging curve of the charged sample was recorded and the discharging curves without bacteria served as the control.

### Physicochemical change of LB medium during and post charging process

The LB medium charged for 15 min or sat idle on the charged samples for 20 min was collected to determine the ORP and pH with the ORP probe (Clean L’eau ORP30, USA) and pH probe (Clean L’eau PH30, USA) following the recommended protocols^67^.

### Physiological changes-morphology of bacteria

The bacteria solution with a concentration of 2–3 × 10^7^ CFU mL^−1^ was treated for 20 min and 180 min with the charged samples. The samples with adhered bacteria were immersed in 2.5% glutaraldehyde overnight and treated with gradient alcohol (10, 30, 50, 70, 90 and 100%) for 10 min each for dehydration before they were dried in vacuum. The samples were then put on a specimen stage prior to SEM. Membrane potential test was also carried out to evaluate the integrity of bacteria membrane (Supplementary Method 6).

### Physiological changes-fluorescent staining of bacteria

The bacteria treated with the charged samples were stained by the LIVE/DEAD® *Bac*Light™ Bacterial Viability Kit (Molecular Probes, Inc., Eugene, OR) to assess the viability. In brief, the live bacteria were stained green and dead bacteria were stained red. 15 min after staining, the samples with bacteria were gently washed with PBS to remove the excess dye and put on a glass slide for observation under an inverted fluorescent microscope (BM-20AYC, BM) with 488/520 nm and 488/630 nm as the excitation/emission wavelengths for green and red fluorescence, respectively. The fluorescent bacteria were also collected and quantitatively analyzed by flow cytometry.

### Physiological changes-intracellular ROS staining

The intracellular ROS levels were determined by the fluorescent probe, 2′, 7′-dichlorodihydrofluorescein diacetate (DCFH-DA, Beyotime, China) which could be deacetylated and oxidized to fluorescent products after crossing the membrane of live bacteria. The bacteria were treated with the charged samples for 20 min and 180 min before 400 μL of DCFH-DA were spread on the sample surface with protection from light for 15 min. The excess dye was removed by PBS and the samples were put on the sample stage under an inverted fluorescent microscope with 488 nm as the excitation wavelength and 520 nm as the emission wavelength. The bacteria treated with 0.1 mM H_2_O_2_ served as the ROS positive group. The bacteria were also quantitatively tested in flow cytometry with the excitation light wavelength set as 488 nm. The *X* Geo mean data of FL1-H was used to evaluate the fluorescence intensity of each group.

### Biocompatibility assessment

The osteoblasts (MC3T3-E1, Chinese Academy of Sciences) were cultured in the Dulbecco’s modified eagle medium supplemented with 10% fetal bovine serum in a humidified atmosphere of 5% CO_2_ at 37 °C and the culture medium was refreshed every other day. To monitor cell adhesion and cytotoxicity, the cells in the logarithmic growth phase were harvested, centrifuged (1000 × *g*), and adjusted to a density of 2 × 10^4^ cells mL^−1^ with the fresh medium before 2.5 mL of the cell solution were seeded onto the surface of the charged sample on a 6-well plate. To quantitatively assess the cell viability, the MTT (Sigma, USA) assay was employed. After incubation for 24, 72, and 120 h on the samples, the medium on the 6-well plate was replaced with PBS to wash the cells and 2.5 mL of the MTT solution (5 mg mL^−1^) were added and cultivated for another 4 h. Dimethyl sulfoxide (DMSO) was used to dissolve formazan. 100 μL of the dissolved formazan were transferred to a 96-well plate and the optical density was determined on a multimode reader (BioTek, US) at 570 nm. Here, the wells with DMSO constituted the negative control group. Fluorescent staining was carried out to evaluate cell adhesion on the samples. At incubation time points of 4 h and 24 h, the cells were fixed with 4% paraformaldehyde in PBS for 30 min, permeabilized with 0.2% Triton X-100 (Sigma, USA) for 15 min, stained with phalloidin-fluorescein isothiocyanate (Sigma, USA) for 30 min, and then stained with 4’,6-diamidino-2-phenylindole (DAPI, Sigma, USA) for 5 min. PBS was used to remove excess reagents and all the procedures were carried out at room temperature. The samples were observed by inverted microscopy with 488 nm/520 nm and 358 nm/461 nm being the excitation/emission wavelengths for green and blue fluorescence, respectively. To determine the intracellular ROS levels, the cells were cultivated on the samples on a 6-well plate with cells treated with 0.5 mM H_2_O_2_ serving as the positive control. After incubation for 4 and 24 h, the cells were rinsed with PBS twice and treated with DCFH-DA for 15 min at 37 °C^68^. The samples with adhered cells were rinsed with PBS and examined by inverted fluorescent microscopy. The green fluorescence represented cells with positive ROS signals.

### Statistical analysis

The data were evaluated by the Student *t* test and SNK test in ANOVA. The data were shown as mean ± s.d. (SD, *n* = 3) and a difference of *P* < 0.05 was considered significant.

### Data availability

All the relevant data are available from the corresponding authors upon request.

## Electronic supplementary material

Supplementary Information
