# Supplementary material for: An antibacterial platform based on capacitive carbon-doped TiO2 nanotubes after direct or alternating current charging
Source: Nat Commun. 2018 May 24;9:2055. doi: 10.1038/s41467-018-04317-2 (PMC5967314; doi:10.1038/s41467-018-04317-2)
Supplement: Supplementary file 1 — Supplementary Information [file 41467_2018_4317_MOESM1_ESM.pdf]

Supplementary Information

An Antibacterial Platform Based on Capacitive Carbon-Doped TiO_2 Nanotubes after Charging with Direct/Alternating Currents

Guomin Wang,[1,a] Hongqing Feng,[2,3,a] Liangsheng Hu,[4] Weihong Jin,[1] Qi Hao,[1] Ang Gao,[1] Xiang Peng,[1] Wan Li,[1] Kwok-Yin Wong,[4] Huaiyu Wang,[1,5,*] Zhou Li,[2,3,*] Paul K. Chu[1,*]

[1] *Department of Physics and Department of Materials Science and Engineering, City University of Hong Kong, Tat Chee Avenue, Kowloon, Hong Kong, P. R. China*

[2] *CAS Center for Excellence in Nanoscience, Beijing Institute of Nanoenergy and Nanosystems, Chinese Academy of Sciences, Beijing 100083, P. R. China*

[3] *School of Nanoscience and Technology, University of Chinese Academy of Sciences, Beijing 100049, P. R. China*

[4] *Department of Applied Biology and Chemical Technology and the State Key Laboratory of Chirosciences, The Hong Kong Polytechnic University, Hung Hom, Kowloon, Hong Kong, P. R. China*

[5] *Research Center for Biomedical Materials and Interfaces, Shenzhen Institutes of Advanced Technology, Chinese Academy of Sciences, Shenzhen, 518055, P.R. China.*

Email adress: hy.wang1@siat.ac.cn (H. Y. Wang); zli@binn.cas.cn (Z. Li); paul.chu@cityu.edu.hk (P. K. Chu).

[a] Guomin Wang and Hongqing Feng contributed equally to this work.

Supplementary Figures.

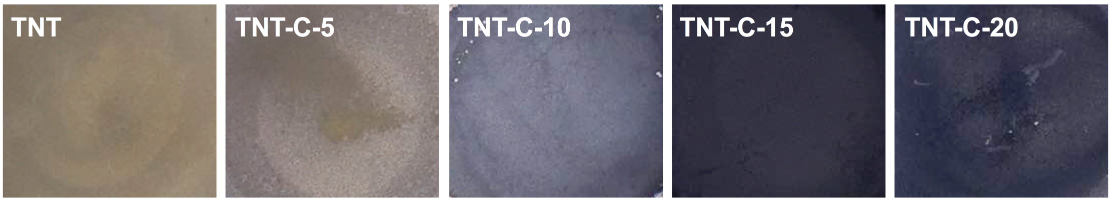

Supplementary Figure 1. Optical images of the samples.

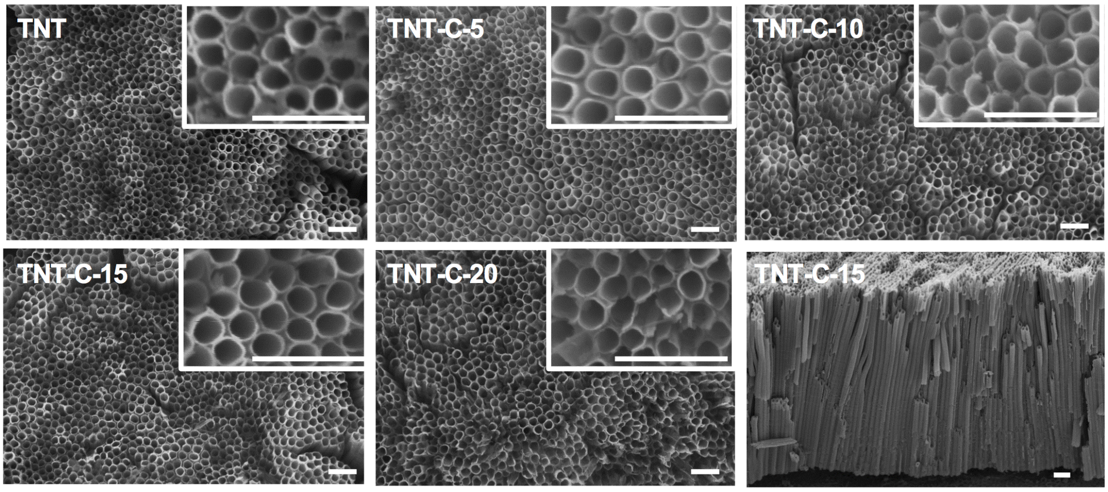

Supplementary Figure 2. SEM images with the insets showing the corresponding enlarged images (Scale bar = 500 nm).

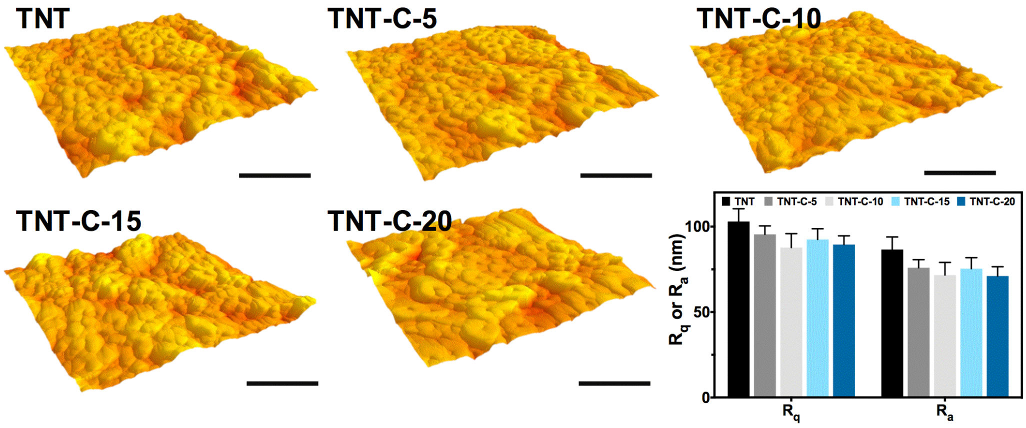

Supplementary Figure 3. AFM images showing the surface morphology of the

samples (Scale bar =500 nm) as well as bar chart showing corresponding roughness. Error bars represent ± SD (n = 3).

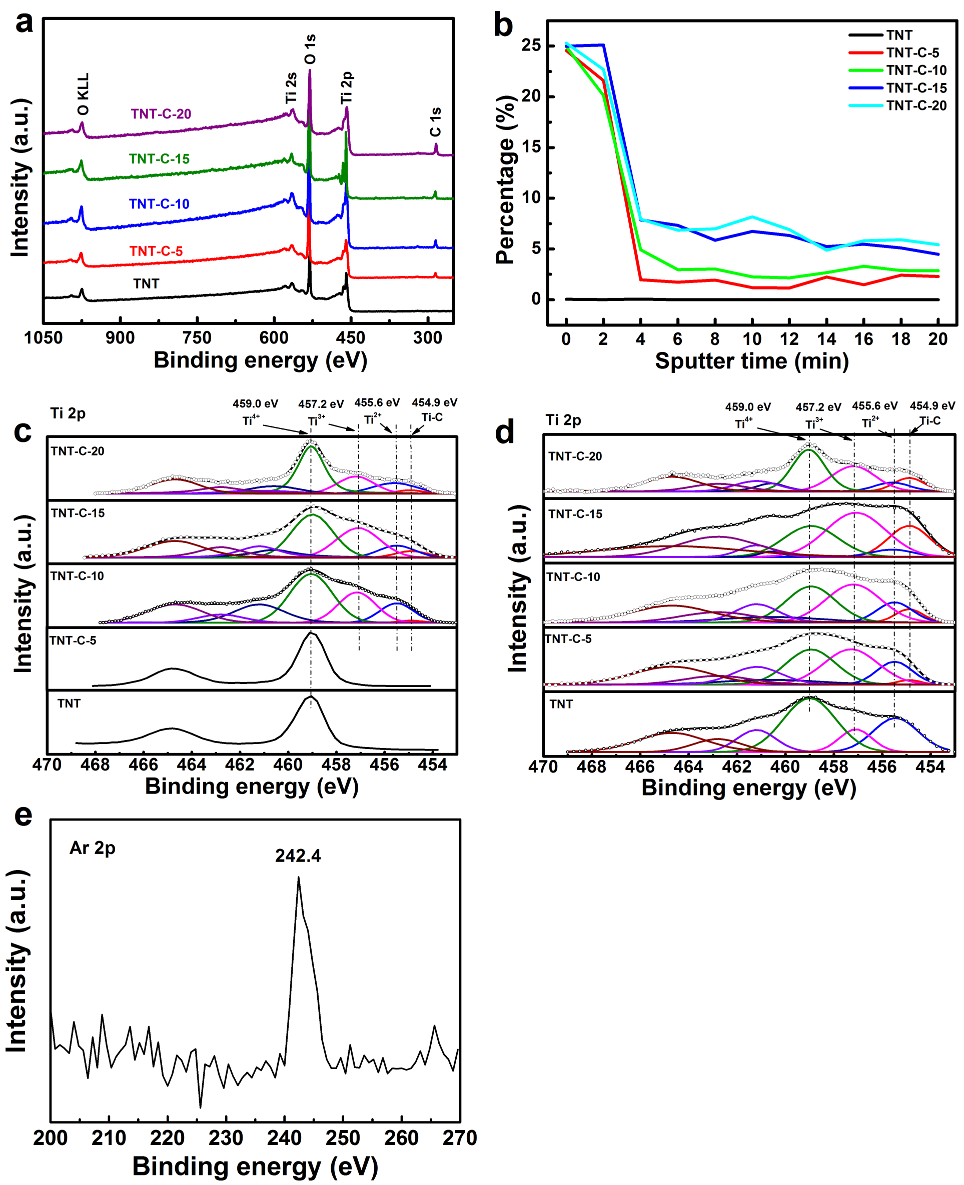

Supplementary Figure 4. XPS results: **a** XPS survey spectra. **b** Corresponding depth profiles showing the C concentrations. High-resolution Ti *2p* spectra acquired **c** from the surface and **d** after sputtering for 6 min. The sputtering rate is approximately 21 nm min^{-1} referenced to SiO_2. **e** Fine spectrum of Ar *2p* for reference.

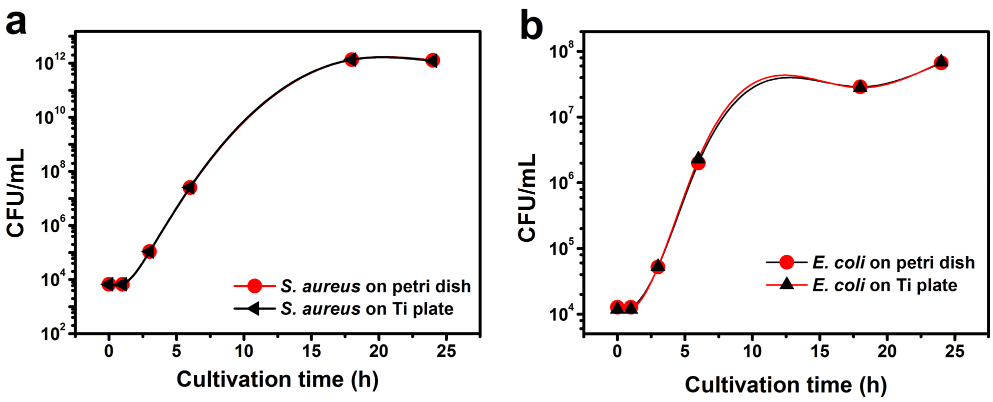

Supplementary Figure 5. Growth curves of **a** *S. aureus* and **b** *E. coli* on the petri dish and Ti plate.

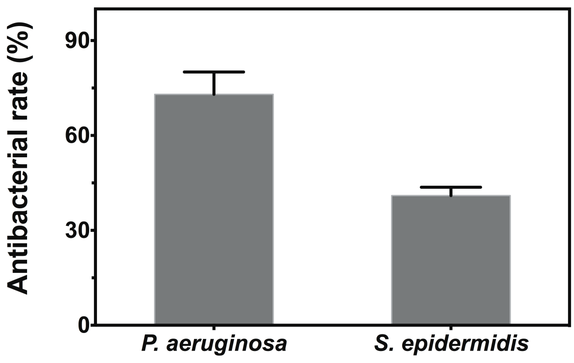

Supplementary Figure 6. Post-charging antibacterial rates of DC+ charged TNT-C-15 on *S. epidermidis* and *P. aeruginosa* after cultivation of 20 min. Error bars represent ± SD (n = 3).

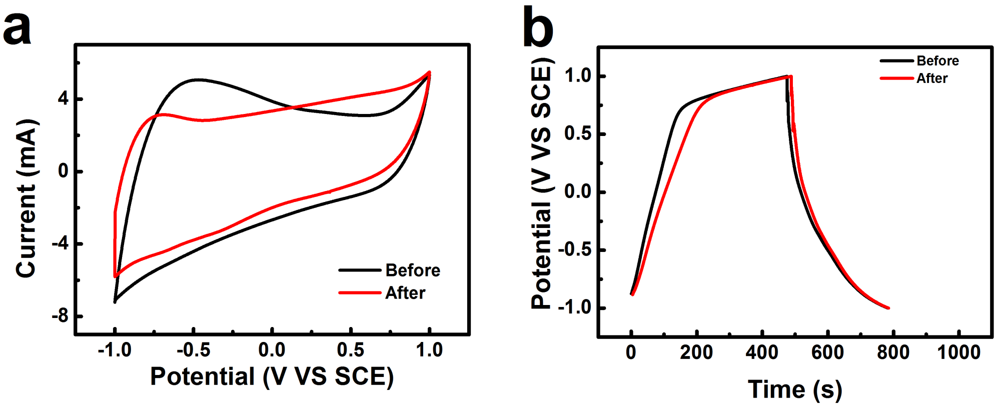

Supplementary Figure 7. Electrochemical performance of TNT-C-15 before and after the antibacterial process: **a** CV and **b** GCD curves acquired at a scanning rate of 100 mV s^{-1} and current density of 2.5 mA cm^{-2}.

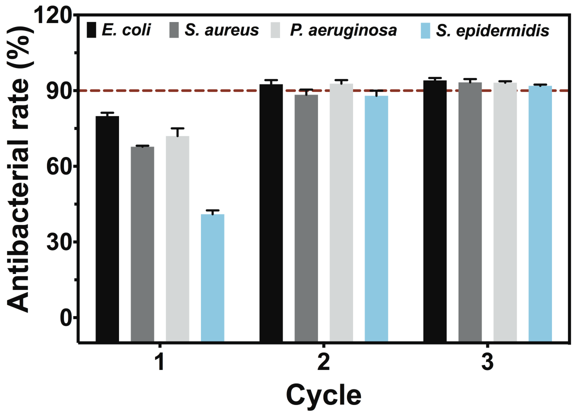

Supplementary Figure 8. Recycling post-charging antibacterial effects of DC+ charged TNT-C-15 on *E. coli*, *S. aureus*, *P. aeruginosa*, and *S. epidermidis*. Error bars represent ± SD (n = 3).

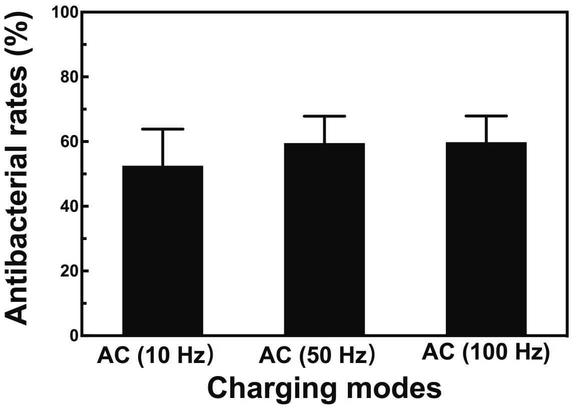

Supplementary Figure 9. Post-charging antibacterial rates of TNT-C-15 triggered by AC at different frequencies after cultivation of 20 min. Error bars represent ± SD (n = 3).

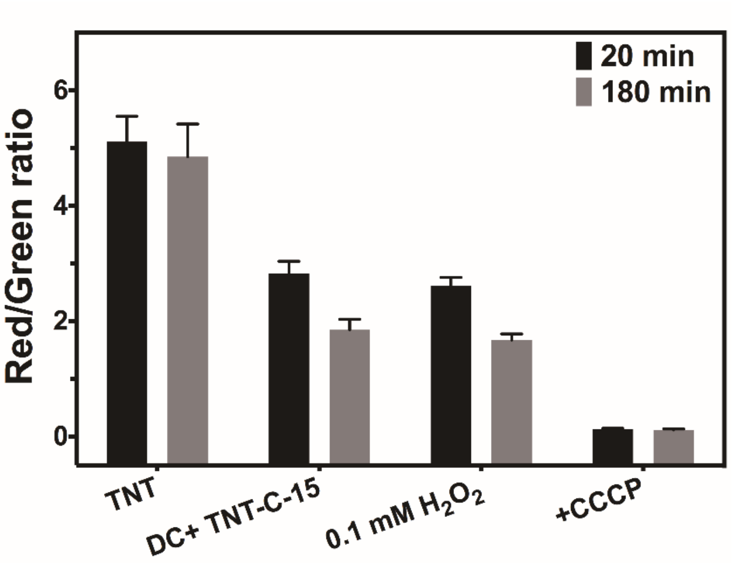

Supplementary Figure 10. Membrane potential of DC+ charged TNT-C-15 group with comparison to TNT and the ROS positive groups. A higher red/green fluorescence intensity ratio means a higher membrane potential. CCCP served as the

positive group of membrane destruction. Error bars represent ± SD (n = 3).

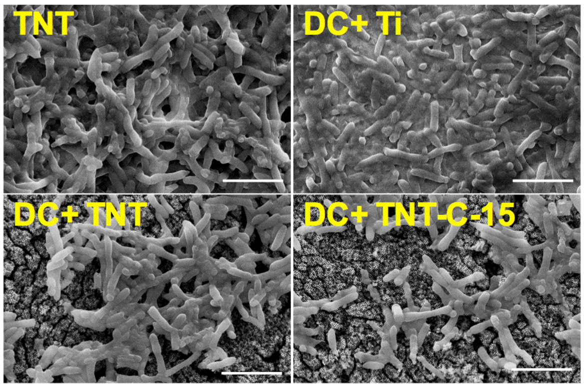

Supplementary Figure 11. SEM images of the biofilms (Scale bar = 5 μm).

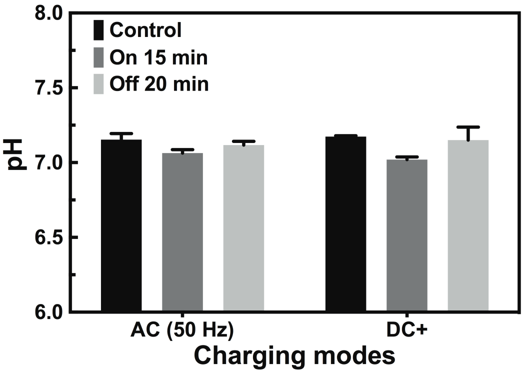

Supplementary Figure 12. pH of the LB medium during and after the charging process. Error bars represent ± SD (n = 3).

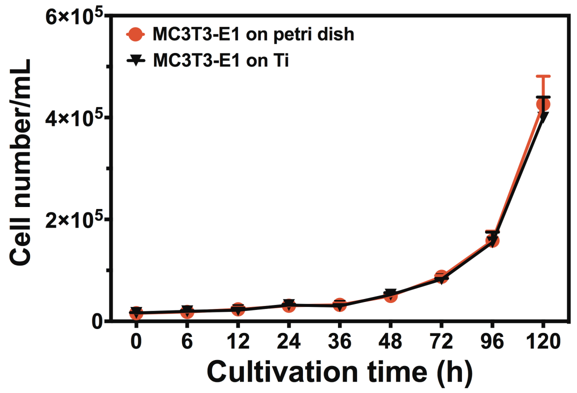

Supplementary Figure 13. Growth curves of MC3T3-E1 on the petri dish and Ti plate. Error bars represent ± SD (n = 3).

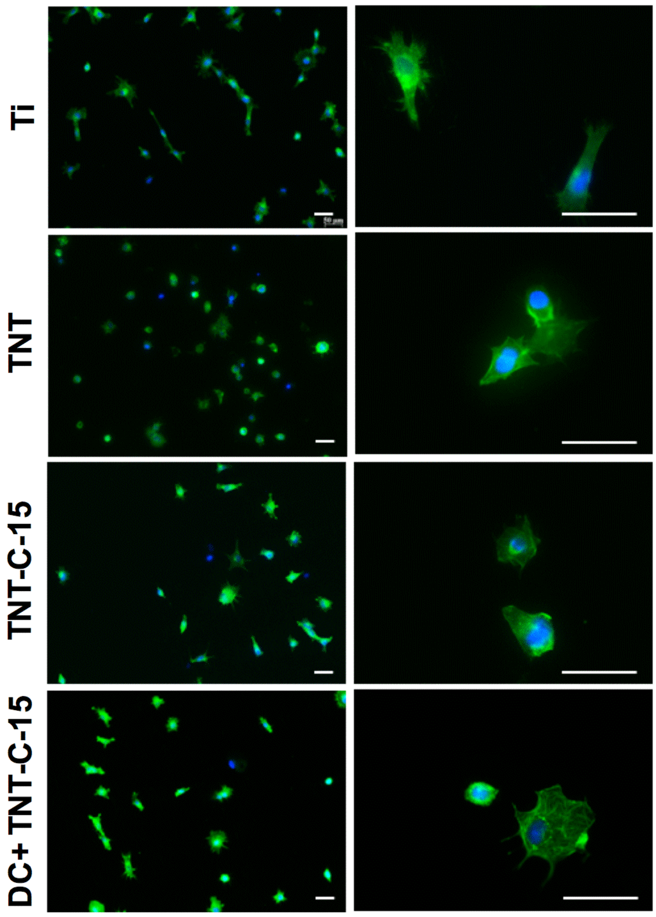

Supplementary Figure 14. Cell morphology of MC3T3-E1 osteoblasts cultivated on different samples for 4 h (Scale bar = 50 μm).

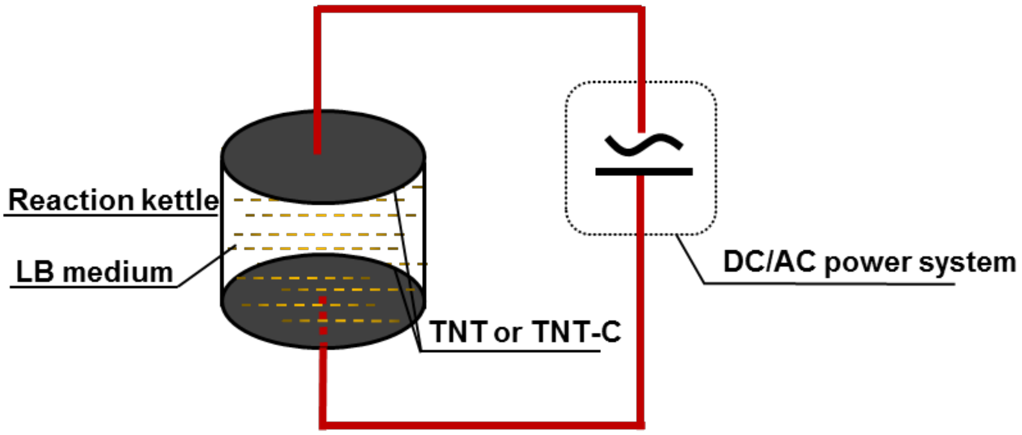

Supplementary Figure 15. Schematic illustration of the DC or AC charging process.

Supplementary Methods.

1. **Detailed description of the charging process with DC and AC.** Before the charging process, two pieces of the TNT-C samples were fixed in the specially designed reaction kettle as shown in Supplementary Figure 15. One piece was fixed to the bottom of the kettle and the other was put on top of the kettle. The space between the two TNT-C pieces were filled with the LB medium and the two samples were connected to the respective power supplier. In the DC charging system (IT6123, ITECH, Nanjing, China), the sample connected to the anode was named DC+ and the sample connected to the cathode was named DC-. After charging with DC at 2 V for 15 min, the samples were removed and put on a petri dish before 400 μL of the bacteria solution were spread on the surface for the following antibacterial process. For the AC treatment, the TNT-C pieces were

connected to the two ends of the AC (FY3200S, FeelTech, Shenzhen, China) power system with no discrimination. In the AC charging system, both charged samples were named AC and used for the subsequent antibacterial tests as mentioned above. With reference to the electrochemical results, the CV curves were acquired between the range of -1~1 V (Fig. 2a) and 2 V was selected as the charging voltage to assess the performance. The post-charging antibacterial rates of TNT-C-15 triggered by AC at different frequencies showed no significant differences (Supplementary Figure 9). 50 Hz was selected as the model AC power supply because it is commonly used.

2. **Antibacterial effects of TNT-C-15 on *Staphylococcus epidermidis* and *Pseudomonas aeruginosa.*** Two additional strains of bacteria (*S. epidermidis*, pAO1 and *P. aeruginosa*, clinical isolate) were included in our study to confirm the capacitance-dependent antibacterial effects of TNT-C-15. In brief, the pure bacteria in LB were cultivated overnight in a rotating shaker at 37 ºC, twice diluted, and cultivated to a concentration of 2-3×10^9 CFU mL^{-1} (OD_{600}=0.3 for *S. epidermidis* and OD_{600}= 1.0 for *P. aeruginosa*). The bacteria solution with a concentration of 2-3×10^5 mL^{-1} was prepared for the subsequent antibacterial test.

3. **Anti-biofilm tests.** *E. coli* were cultivated on various samples up to 48 h. During the process of bacteria cultivation, the samples except TNT control were charged every 8 h. In the quantitative analysis, the specimens were gently rinsed in PBS, stained by 0.1% crystal violet for 20 min, rinsed in a deionized water bath, and the bound crystal violet was eluted by 1 mL of 100% alcohol. Afterwards, the

optical density of eluates was determined on a multimode reader (BioTek, US) at 590 nm [1]. In the qualitative analysis, the samples with adhered bacteria were fixed and dehydrated prior to SEM observation. Besides, the biofilms were stained with the LIVE/DEAD® *Bac*Light™ Bacterial Viability Kit (Molecular Probes, Inc., Eugene, OR) before the 3D morphology was examined by confocal scanning laser microscopy.

4. **Cyclic antibacterial tests.** After each cycle of post-charging treatment, the bacteria were collected from the samples and the samples were recharged for another 15 min. Afterwards, the pre-collected bacteria were again spread on the charged samples to evaluate the cyclic antibacterial effect. The CFU counting method was used to quantitatively calculate the antibacterial rates and the detailed antibacterial procedures are described in the **Methods** section.
5. **Recyclable platform as capacitive materials.** After the first antibacterial process, the samples were cleaned ultrasonically in acetone, alcohol, and deionized water, and dried in nitrogen to remove the remained bacteria. CV was carried out from -1 V to 1 V at a scanning rate of 0.1 V s^{-1} and GCD tests were performed at a constant charging current of 2.5 mA cm^{-2}.
6. **Membrane potential test.** The membrane potential of the bacteria was measured with a membrane potential kit (B34950, Invitrogen, USA). The bacteria treated with carbonyl cyanide m-chlorophenyl hydrazone (CCCP) served as the positive group for membrane destruction. After the post-charging treatment, 4 μL of $DiOC_2(3)$ (3,3′-diethyloxacarbocyanine Iodide, 3 mM) were added to the sample

and after 15 min, the bacteria were collected and assayed by flow cytometry. An excitation wavelength of 488 nm was used to excite $DiOC_2(3)$ and the green and red fluorescence was monitored simultaneously using the 530 nm and 610 nm band-pass filters, respectively. The degree of membrane depolarization was characterized by the red/green fluorescence ratio [2].

Supplementary References

1. Epstein, AK., Wong, T-S., Belisle, RA., Boggs, EM., Aizenberg, J. Liquid-infused structured surfaces with exceptional anti-biofouling performance. *Proceedings of the National Academy of Sciences* **109**, 13182-13187 (2012).

2. Novo, DJ., Perlmutter, NG., Hunt, RH., Shapiro, HM. Multiparameter Flow Cytometric Analysis of Antibiotic Effects on Membrane Potential, Membrane Permeability, and Bacterial Counts of Staphylococcus aureus andMicrococcus luteus. *Antimicrobial agents and chemotherapy* **44**, 827-834 (2000).
